# Human Healthcare and Its Pharmacy Component from a Safety Point of View

**DOI:** 10.3390/pharmacy12020064

**Published:** 2024-04-07

**Authors:** Natalia Tkachenko, Ostap Pankevych, Tamara Mahanova, Bohdan Hromovyk, Roman Lesyk, Lilia Lesyk

**Affiliations:** 1 Department of Pharmacy Management and Economics, Zaporizhzhia State Medical and Pharmaceutical University, 26 Maiakovskoho Ave., 69035 Zaporizhzhia, Ukraine; tkachenkonat2@gmail.com (N.T.); maganova.tv@zsmu.zp.ua (T.M.); 2 Department of Organization and Economics of Pharmacy, Danylo Halytsky Lviv National Medical University, 69 Pekarska, 79010 Lviv, Ukraine; pankevych.lviv@gmail.com (O.P.); hromovyk@gmail.com (B.H.); 3 Department of Pharmaceutical, Organic and Bioorganic Chemistry, Danylo Halytsky Lviv National Medical University, 69 Pekarska, 79010 Lviv, Ukraine; dr_r_lesyk@org.lviv.net; 4 Department of Business Economics and Investment, Institute of Economics and Management, Lviv Polytechnic National University, 5 Metropolian Andrey Str., Building 4, 79005 Lviv, Ukraine

**Keywords:** safety, security, public health, healthcare, pharmaceutical

## Abstract

Healthcare plays a crucial role in public and national safety as a significant part of state activity and a component of national safety, whose mission is to organize and ensure affordable medical care for the population. The four stages of the genesis of healthcare safety development with the corresponding safety models of formation were defined: technical, human factor or security management, systemic security management, and cognitive complexity. It was established that at all stages, little attention is paid to the issues of the formation of the pharmaceutical sector’s safety. Taking into account the development of safety models that arise during the four stages of the genesis of safety science, we have proposed a model of the evolution of pharmaceutical safety formation. At the same time, future research is proposed to focus on new holistic concepts of safety, such as “Safety II”, evaluation and validation methods, especially in the pharmaceutical sector, where the development of this topic remained in the second stage of the evolution of science, the search for pharmaceutical errors related to drugs.

## 1. Introduction

Healthcare plays a crucial role in public safety as a significant part of state activity and particularly as a component of national safety, whose mission is to organize and ensure affordable medical care for the population. The ability of the state to protect national interests in the field of healthcare from possible threats and to ensure the realization of the human right to life and health, medical assistance and medical insurance to eliminate dangers that threaten life and health should be understood as vital to safety [1].

At the same time, issues such as ensuring the medical, social, and economic efficiency of healthcare institutions as well as practical sanitary preventive activities, pharmaceutical safety, the safety of medicinal products, and epidemiological and ecological control are related to the problem of healthcare safety. Therefore, the question of the development of the healthcare system as a structural element of national safety has not lost its relevance. Not only are there concerted efforts from healthcare professionals, patients, consumers, regulatory bodies, and politicians, but also there has been an implementation of the processes of providing medical and pharmaceutical care from the point of view of safety science [2]. Safety science is the process of generating knowledge about safety-related phenomena, processes, events, etc., and its conceptual tools, including the development of concepts, theories, principles, and methods to understand, evaluate, communicate information, and manage safety [3].

The purpose of the work was to carry out a scientific study of safety issues in the «human–healthcare–pharmacy» system, to reproduce the genesis of development and the main approaches to its formation, with the further determination of problematic aspects and the justification of current directions of scientific research.

The research hypothesis assumes that establishing pharmaceutical safety as a system component, as mentioned earlier, is related to healthcare safety and the stages of development of safety science.

## 2. Materials and Methods

General scientific methods of cognition were used: analysis and synthesis, abstraction, deduction, modeling, content analysis, and generalization. The materials were scientific publications from the bibliometric database Google Scholar and the electronic Medline (PubMed) electronic search system from 1930 to 2023. The following keywords mesh terms were chosen: “safety”, “security”, “public health”, “healthcare”, and “pharmaceutical”.

**Figure 1:**
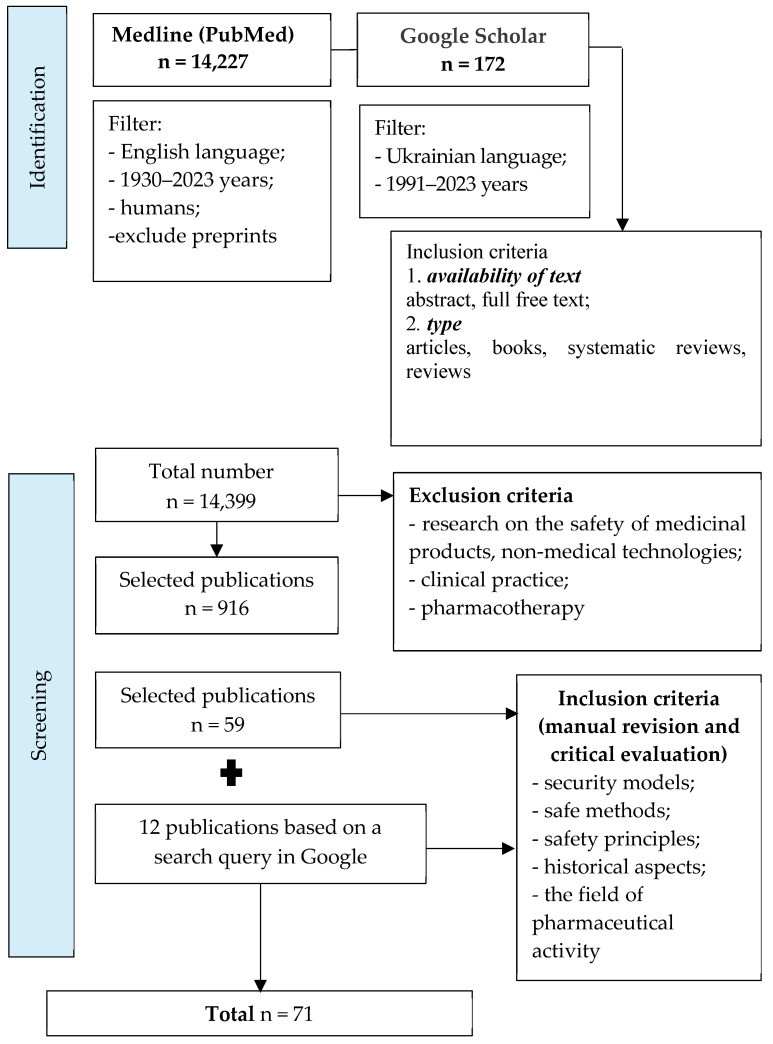
The process of selection of publications.

Through content analysis and the systematization of the obtained results, 916 references were selected from 14,399 publications that met the following criteria: availability of text—abstract, full free text; type—articles, books, systematic reviews, and reviews. During further analysis of the selected literature to achieve the goal of the work, we found 71 publications. The publication selection process is presented as a corresponding diagram of the PRISMA recommendations (PRISMA flow diagram), shown in Figure 1. The search for articles in these databases was determined by the specifics of the given question (theoretical and practical aspects of safety in the pharmaceutical sphere) and its insufficient theoretical development. Both software search tools and manual search with subsequent critical evaluation of articles (Google Academy and Google search engine) were used for maximum coverage of results.

## 3. Results

Safety science has its history and its own retrospective overlap periods, which first appear in the work of Hale and Hovden [4]. Nowadays, four stages of its genesis can be conditionally distinguished: the stage of technologies (1770–1960), the human factors or safety management (1960–2000), the systemic safety management stage (2000–2015), and the cognitive complexity stage (2015–present).

Technology remained at the center of safety measures from the beginning of the Industrial Revolution until the middle of the 20th century. Their unreliability and the risk of injury posed a primary threat to workers and consumers who received low-quality products [5]. This direction of the development of safety science was called the «technological stage», where the Domino model was developed to detect and overcome threats (safety formation) [6].

According to the intention of its author, the analysis of events was aimed at finding a particular component, a “bone”, which, according to the Domino principle, caused a dangerous situation in a linear sequence of processes. Despite the simplicity of the model, it remained advanced in forming safety at that time, including in the healthcare field. At the same time, the scientific study of the interaction of «expert-technology» was focused only on efficiency and productivity. Such systems were offered only limited protections. Therefore, further reforms were necessary [7]. While progressing and facing several human-caused disasters, the Domino model lost its relevance.

The one-sided view of technology, process automation, and workers has changed. Previously, workers were simply part of an undefined “the poor” group. Now, they are a group that deserves attention. People have started to be considered imprecise and unstable, and the causes of disasters and accidents have begun to be considered failures in the work process and the stress of workers who overcome these failures [6].

In healthcare, the emphasis on professionals has led to the consideration of them as reliable subjects with the potential for unreliability and with the intent to contribute to intentional or unintentional errors [5]. In the pharmaceutical sector, this contributed to the development of national normative legal acts that regulated certain aspects of pharmaceutical activity (manufacturing technology in a pharmacy, pharmaceutical storage conditions, sanitary standards, etc.).

Scientists began to consider technology dually as an inseparable and undesirable component of care simultaneously [8]. Occupational hygiene, the science of identifying, measuring, and controlling potentially harmful workplace exposures, occupational medicine, toxicology, and epidemiology continued to grow, as did disciplines associated with safety design and engineering [7]. This second stage of the evolution of safety was called the “stage of the human factor”.

Considering the safety of the healthcare system from the position of the object of directed action, the safety of patients has always remained as the main priority. Moreover, although mentions of the organization of patient safety measures in scientific sources can be found as early as the 1950s [9], for the first time, special attention was paid to the problem of personal safety only at the end of the 20th century when in the Human Development Report Office of the United Nations Development Programme (1994) identified seven main components of human security [10]:

− economic security—guaranteed basic income;
− food security—constant physical and economic availability of basic food products;
− health security—equal access to medical services without discrimination based on the availability of financial resources, place of residence, type of disease, social and racial affiliation;
− environmental safety—availability of high-quality drinking water, clean air, sustainable land use, prevention of man-made and natural emergencies;
− personal security—freedom and protection from physical violence and threats, protection from threats to oneself;
− community security—protection through belonging to a group—family, community, organization, racial or ethnic group—that can provide a cultural identity and a reassuring set of values;
− political security—the protection of basic human rights and freedoms.

And only since the 2000s have systematic efforts been made to reduce deaths and injuries in hospitals [9]. Thus, at the end of the 20th century, patient safety was formed to develop emergency response services (helicopter intensive care system), surgical interventions and preoperative support of patients, and the safety of injection procedures in countries with a developing economy [11,12,13,14].

At this time, a new model of «Swiss cheese» or «cumulative action» emerged, which was formalized initially in the 1990s [15]. According to this, any event is a linear causality, preceded by a combination of dangerous actions and conditions, visualized as pieces of cheese layered on each other, considering individual characteristics of the specialist, team, and organizational factors. Due to the property of the linearity of the “Swiss cheese” model, as in the previous stage of safety, the possibility of reverse identification of causes was preserved, where the beginning of the countdown is the final adverse event.

This approach is still actively used in healthcare [16]. Based on the «Swiss cheese» model, the concept of achieving safety was formed: the “hazard-barrier-target”. The “Swiss cheese” model shows how a series of safety barriers can break down and allow hazards to propagate due to loss events. In this model, barriers are represented by slices of Swiss cheese, with the holes representing failures in the barrier. If an initiating event occurs and the holes in the barriers align, a loss event will occur [17]. This period is called “safety management” [18,19]. Moreover, although it is rarely singled out in the scientific literature, it can be considered reductionist.

The pharmaceutical sector introduced five concepts: good pharmaceutical practices (GxP), pharmaceutical care, the seven-star pharmacist, social pharmacy, and social responsibility [20,21]. The critical professional role of the pharmacist has evolved from drug-centered to patient-centered practice. At the same time, pharmaceutical care was not isolated from other healthcare services and was provided in cooperation with patients, doctors, nurses, and other providers of medical services. However, drug safety remains an urgent safety issue in pharmacy.

At the beginning of the twentieth century, according to a retrospective analysis by Hale A. and Hovden J. [22], the development of the third stage of safety—systemic safety management—began. It turned out that in complex systems in which technical, human, and organizational factors interact closely at multiple levels and exhibit complex behavior, the occurrence of dangerous situations cannot be explained by superficial linear cause-and-effect relationships. Since then, the global philosophy of sociotechnical systems has acquired its practical significance and gained recognition and an audience beyond the boundaries of the social sciences [23].

According to [24], the safety of the healthcare system started to be considered a systemic phenomenon, emphasizing the physical and economic access to medical services of vulnerable and disenfranchised sections of the population in the conditions of insurance medicine. The continuation of the specified vector resulted from many publications that testified to the importance of medical care in the overall system of safety in the healthcare industry and national security [25,26]. An essential step in this process was the attempt to determine the criteria for assessing the safety status, planning, and reallocation of the limited resources of the system based on accurate patient data [27].

In the following years, the topic of scientific works on patient safety continued in the selected vector separately, touching on specific issues not of a systemic nature: the safety of women in labor [28], transplantology [29], the reduction in risks associated with the medical field [30], the availability of medical care for patients with cardiovascular diseases [31], planned vaccination and the prevention of infectious diseases [32], the development of a set of recommendations and principles for the design of a hospital facility focused on patient safety [33], and combating psychological violence against patients in the primary care chain [34].

Achieving the goal of increasing patient safety has led to the formation of many concepts and models [35,36], at the center of which are scientists’ attempts to understand and teach medical professionals the everyday features of work in complex socio-technological systems. These trends turned into attempts in the healthcare sector to borrow and implement a safety management system, a proactive safety culture, and methods of their constructive evaluation from high-risk industries such as aviation, oil and gas, and the chemical industry, including through the analysis of the evolutionary safety model “Safety I” [37].

The International Classification of Patient Safety (ICPS) published a standardized set of concepts and terms combined in a conceptual framework consistent with key concepts [36,37] and organized the main adverse events related to patient safety in socio-technical systems. According to ICPS, safety reduces the risk of unnecessary harm to an acceptable minimum, and system improvement or the culture, processes, and structures directed toward preventing system failure and improving safety and quality are the results [38]. Risk management in such systems is aimed at identifying causes and factors.

ICPS development was first identified as a key initiative of the WHO Global Alliance for Patient Safety in 2005 [39]. However, the formation of a conceptual framework, taxonomy and the borrowed concept of safety management from industrial systems, “Safety I”, turned out to be insufficient for large socio-technical systems such as the healthcare system [40], which led to the creation of an alternative new concept of safety and its practical methods: “Safety II” [5,41].

The new concept, first of all, changed the attitude toward safety. Thus, in “Safety I” safety is defined as a state where as few processes as possible will go wrong due to technical, human, and organizational reasons, and the human resource is considered responsible for the violation or actually as a source of danger. However, the activity of a specialist almost always goes well, which is overlooked in “Safety I”. The main reason for this is the possibility of adaptation, the adjustment of processes, and the work of specialists in response to given conditions [41].

Safety II may be seen as a positivist version of occupational health and safety, in contrast to safety thinking (Safety I), in which safety is seen as the ability to navigate and cope with the stress and high-pressure situations inherent in the modern and complex world of work. This concept focuses not on imposing standards and rules but on recognizing and promoting the human ability to work safely and successfully without rigid adherence to rules.

As the healthcare system develops, processes become more complex and these settings «on the ground» become increasingly crucial for the successful implementation of assigned tasks. The critical property of safe systems within the Safety II concept is resilience to conditions that create errors. Resilience may be defined as being able to perform as needed under various conditions and responding appropriately to disturbances and opportunities [42].

A person is considered a resource necessary for a flexible and resilient system. Safety management consists of constantly predicting the development of events. As the search objective changes, risk management aims to understand the condition where performance variability may become difficult or impossible to control. In this case, the attribution of a mistake to a single person is eradicated from the system, the principle of establishing the culprit is the end of the investigation of organizational functions, norms, and behavior [5], and the punitive culture that forces the employee to feel in danger is nullified. The most important thing is that the system eliminates the principle of correcting what is faulty. After all, ignoring this allows the system to achieve resilience, work successfully, and prevent errors from occurring.

This perspective of safety management is embodied in “Safety II”, which was enthusiastically accepted in the healthcare system [43]. This focus on resilience necessitated the formation of a proactive safety culture in the healthcare system, where the specialist, as a resource of the system, is a decisive factor in processes that go correctly.

The first mentions of safety culture are found as early as 1991 [44,45]. In these works, the safety culture was understood as the product of individual and group values, attitudes, perceptions, competencies, and patterns of behavior that determine the commitment to and the style and proficiency of an organization’s safety management [44]. In a systematic review, Weaver S.J. et al. point out that developing a culture of safety is a core element of many efforts to improve patient safety and care quality in acute care settings [46], and improving patient safety culture should include all stakeholders, like policymakers, healthcare providers and those responsible for medical education [47]. Despite the complexity of the conceptual basis, this approach to the analysis and support of the proper work of health professionals as a critical link in the formation of system safety continued to be updated and expanded.

Starting with articles on improving patient safety culture [48], ensuring optimal sleeping and working conditions for doctors and nurses, reducing the level of injury to specialists, organizing occupational hygiene, creating new training designs, including in the context of the informatization of the healthcare system and the development of new interfaces, adjusting the duration of the shift following the needs of specialists for the interaction and organization of teamwork, preventing emotional and mental burnout and optimal staffing is paramount for healthcare systems [49]. It is worth noting that only a few scientists noted pharmacists’ role in shaping the safety of the healthcare sector [50].

However, the concept of “Safety II” still has many ambiguities that need to be resolved: the proof of concept, safety measurement and evaluation, research validation and the evidence of its effectiveness, which cannot be based on indicators borrowed from “Safety I” [19]. The methodologies “Appreciative Inquiry methodology”, and “Positive Deviance” are considered promising, which, in theory, should stimulate the improvement of the system by identifying “useful adaptations” or “positive deviations” [22], and assist in the application of new approaches to assess the competences of specialists at the workplace, which are similar in nature to the stability properties and can be used in the future [51].

## 4. Discussion

As indicated, the main issue is the transfer of the «Safety II» theory to healthcare without losing other important principles of healthcare, because the system’s safety is one of the aspects of the quality of medical care. At the same time, timeliness, efficiency, fairness, and patient orientation are also important. This is reflected in some subsequent work, where the involvement of patients is recognized as an essential step in establishing the system and patient safety, and the combination and integration of person-oriented strategies with organization-oriented strategies is still in the design stage [52,53].

In particular, this barrier of abstractness of ideas and principles creates the prerequisites for a slow transition to systemic security management. Furthermore, although Hollnagel E. claims that we should move away from determining the causes of accidents using the principle of ETTO (efficiency-thoroughness trade-off), the issue of error detection continues to be heavily emphasized in the scientific works of scientists [54,55], including the pharmaceutical sector, where, as at the previous stage, the primary attention when understanding safety is focused on drugs [38] and pharmacists’ mistakes [56,57]. At the same time, safety was defined as a systematic study of the negative impact of drugs and devices on humans at all stages of the drug life cycle [58,59].

**Figure 2:**
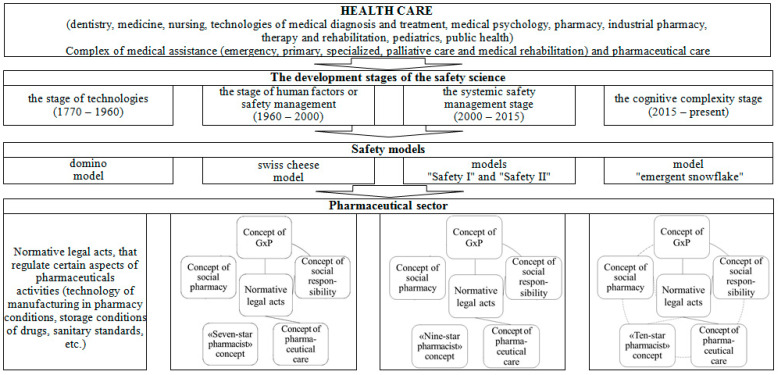
The model of the evolution of formation of pharmaceutical safety.

Considering the development of safety models that arose during the four stages of the genesis of safety science, we proposed a model of the evolution of the formation of pharmaceutical safety (Figure 2). The technology stage is characterized by the development of normative legal acts that regulate certain aspects of pharmaceutical activity (manufacturing technology in a pharmacy, storage conditions for medicinal products, sanitary standards, etc.). As mentioned above, the concept of GxP, the concept of pharmaceutical care, the “Seven-star pharmacist” concept, the concept of social pharmacy, and the social responsibility concern have been introduced at the human factor stage. The developed normative legal acts relate to the concepts of GxP, pharmaceutical care, and social responsibility. At the same time, pharmaceutical organizations apply the concepts of GxP and social responsibility in an interconnected manner.

At the stage of system safety management, the “Seven-star pharmacist” concept was replaced by the “Nine-star pharmacist” concept. The rest of the changes are related to the normative support of the concepts of GxP, pharmaceutical care, and social responsibility, as well as the theoretical content of other concepts. A similar development still characterizes the stage of cognitive complexity. Here, the concept of the “Nine-star pharmacist” was replaced by the concept of the “Ten-star pharmacist.” We believe developing regulatory and legal acts for all concepts and their relationship during implementation in pharmaceutical practice will be a perspective for developing pharmaceutical safety.

It is worth noting that in recent years, more and more attention has been paid to the formation of safety in IT technologies, telemedicine [60], and telepharmacy. This aspect can be considered a new stage in safety management systems, adding to the formation of holistic safety and patients. Some scientists suggest considering safety as a complex of organization, people, and environment, as understood by these healthcare facilities [61] and environmental intelligence [62]. However, these directions are still closer to the reductionist approach mentioned above. The provision and training of healthcare professionals on safety culture issues is considered significant [63], but pharmacists are again neglected [62]. In addition, the issues of digital information safety, which covers all links of medical and pharmaceutical care in socio-technical systems, including the same environment and patients who are its users, are being updated [64,65].

At the same time, scientific studies that raised questions about the conceptual foundations of the formation of the pharmaceutical safety system [66] left the safety indicators of drugs as basic. Other scientific works on pharmaceutical safety [67] do not take into account the pharmacist, who is an obligatory member of the multidisciplinary teams in healthcare and performs ten prominent professional roles according to the concept of the “Ten-star pharmacist”: a caregiver, a decision-maker, a communicator, a manager, a life-long learner, a teacher, a leader, a researcher, an entrepreneur and an agent of positive change [68]. A pharmacist treats patients at the same level as a doctor [69]. The third stage of the development of pharmaceutical safety will provide a set of measures to minimize the risks associated with the circulation of pharmaceutical products in the context of the safety of the population, pharmaceutical companies, and the environment, as well as the defense and economic independence of a country [70]. However, its practical implementation requires further systematic and comprehensive research.

Safety science is actively developing, and today scientists are talking about a new stage, the stage of cognitive complexity, in which specialists act as carriers of information about how best to reach a safe zone. Furthermore, safety is an emergent property of a complex adaptive system [71]. In this context, the concept of pharmaceutical safety also changes.

At the same time, future research should be focused on new concepts, such as holistic concepts of safety, such as “Safety II”, and evaluation and validation methods, especially in the pharmaceutical sector, where the development of this topic remained at the second stage of the evolution of science—the search for pharmaceutical errors related to drugs.

The limitations of this work include a lack of searching in other specialized databases, as the search only included results in English or Ukrainian, only full-text publications, and lacked search sensitivity. It should also be noted that the terms MeSH used in the article still enabled the inclusion of some related concepts (“pharmaceutic”[All Fields] OR “pharmaceutics”[All Fields] OR “pharmaceutical preparations”[MeSH Terms] OR (“pharmaceutical”[All Fields] and so on), which reduces the probability of missing essential studies. Since the topic of safety is extensive and the search keywords cover significant concepts, our task was, to a greater extent, a general analysis of the safety situation in the pharmaceutical field with an outline of the main concepts borrowed and a search for directions for subsequent research.

## 5. Conclusions

The analysis of the scientific literature on safety issues in the «human–healthcare–pharmacy» system allows us to determine four stages of the genesis of its development with the corresponding safety models of formation, technical, human factor or safety management, system safety management, and cognitive complexity.

It was established that at all stages, little attention is paid to the issues of the formation of the pharmaceutical sector’s safety. Considering the development of safety models that arose during the four stages of the genesis of safety science, we have proposed a model of the evolution of pharmaceutical safety formation. At the same time, future research is proposed to focus on new holistic concepts of safety, such as “Safety II”, and evaluation and validation methods, especially in the pharmaceutical sector, where the development of this topic remained in the second stage of the evolution of science, the search for pharmaceutical errors related to drugs.

## Data Availability

Data are contained within the article.
